# Retardance and depolarization of brain white matter as markers for intraoperative delineation of brain tumors: experiments and simulations

**DOI:** 10.1364/BOE.577075

**Published:** 2025-11-26

**Authors:** Meishu Wang, Sooyong Chae, Vanesa Lukinsone, Théotim Lucas, Omar Rodríguez-Nuñez, Éléa Gros, Christopher Hahne, Theoni Maragkou, Richard McKinley, Philippe Schucht, Tatiana Novikova

**Affiliations:** 1 MOE Key Laboratory of Optoelectronic Imaging Technology and System, Beijing Institute of Technology, Beijing 100081, China; 2 LPICM, CNRS, Ecole polytechnique, IP Paris, Palaiseau 91120, France; 3 Institute of Atomic Physics and Spectroscopy, Faculty of Science and Technology, University of Latvia, Riga, Latvia; 4 Department of Neurosurgery, Inselspital, Bern University Hospital, University of Bern, 3010 Bern, Switzerland; 5 Institute of Tissue Medicine and Pathology, University of Bern, 3008 Bern, Switzerland; 6 Graduate School for Cellular and Biomedical Sciences, University of Bern, 3012 Bern, Switzerland; 7 Support Centre for Advanced Neuroimaging, Institute of Diagnostic and Interventional Neuroradiology, Inselspital, Bern University Hospital, University of Bern, 3010 Bern, Switzerland; 8 Department of Biomedical Engineering, Florida International University, Miami, FL 33174, USA; 9 3120225325@bit.edu.cn; 10 tatiana.novikova@polytechnique.edu

## Abstract

An accurate distinction between brain tumors and tumorless brain tissue is crucial for effective surgical resection. Polarization-sensitive optical imaging exploits birefringence differences, offering contrast between the optically anisotropic white matter of the tumorless brain and the optically isotropic brain tumor tissue. However, crossing brain fiber bundles within tumorless brain tissue may also erase such optical anisotropy. We use a polarized Monte Carlo algorithm to model backscattered wide-field Mueller matrix images of the optical phantoms of the brain's white matter. We compare the impact of fiber bundle crossing and the presence of an optically isotropic subsurface tumor across varying depths to mimic brain tissue removal during neurosurgery. The simulation results demonstrate that the depolarization dependence on depth may serve as a decisive parameter to distinguish the tumor and fiber bundles crossing zones, as the values of linear retardance drop in both zones, whereas the depolarization values become smaller in the tumor zone.

## 1. Introduction

During brain tumor surgery, the identification of the precise location of the tumor boundary is very important [1,2], as it can help neurosurgeons remove the tumor zone completely and reduce the probability of tumor recurrence without excessively damaging tumorless brain tissue [3]. Knowledge about the precise of location of this tumor-brain interface is essential for respecting the tumor-free brain and, hence, it helps to preserve neurological function and patient’s quality of life. Pre-operative magnetic resonance imaging (MRI) is commonly used to determine the presence and approximate boundaries of a tumor, but detecting the glioma and glioblastoma tumor borders *in vivo* remains one of the main challenges of modern neurosurgery [4]. Intraoperative ultrasound is widely used but often suffers from insufficient spatial resolution, resulting in low contrast at tumor margins and inaccuracy in estimating the extent of resection and the residual tumor volume [5,6]. Fluorescence-guided surgery, such as 5-ALA-based visualization, can visualize high-grade gliomas, but requires exogenous agents and may not highlight tumor infiltration regions [7]. Intra-operative MRI provides high resolution imaging during neurosurgery, but is expensive and time-consuming, making it impractical for repetitive use. These constraints underline the need for alternative, *in vivo*, label-free, real-time, and near-continuous imaging approaches that are sensitive to microstructural differences between tumor and tumorless brain tissue [8].

Polarization-sensitive optical imaging has emerged as a promising solution [9,10]. It exploits the changes in polarization states of backscattered light caused by the structural and optical properties of biological tissues. In particular, birefringence, which arises from the anisotropic organization of fibrous tissues [11,12], such as brain white matter, introduces phase retardation and alters the polarization state of backscattered light. Conversely, tumor tissue typically lacks such organized fiber structures and behaves as an optically isotropic medium, resulting in reduced or absent birefringence [13,14]. This intrinsic difference offers a contrast mechanism based on polarimetric parameters such as linear retardance, depolarization, and optical axis orientation. Our proof-of-concept studies demonstrated that wide-field imaging Mueller polarimetry is sensitive to the anisotropy of the refractive index of white matter of tumorless brain related to the presence of densely packed brain fiber bundles [15], which are destroyed by brain tumors. It suggests using the birefringence of tumorless brain white matter as an optical marker for delineation of the tumor border [16,17]. However, the loss of retardance can not only be observed in the tumor zone. The spatial orientation distribution of anisotropic structures can affect polarization parameters such as depolarization, linear retardance, and fast axis orientation, which are very sensitive to the crossing [18,19] and inclination [20]. Therefore, it is crucial to correctly distinguish the polarization properties of tumors and tumorless tissues.

Monte Carlo simulations offer a powerful and widely validated modeling tool to explore and quantify these effects in complex structures [21–25]. It can stochastically simulate photon propagation through scattering and absorbing media, making them especially suitable for studying light-tissue interactions in biological environments. Polarization-sensitive Monte Carlo models have been successfully applied to birefringence media, yielding insights that align with experimental observations in polarization-sensitive optical coherence tomography (PS-OCT) and Mueller polarimetry [26,27]. These models have been extensively applied to investigate photon migration, optical penetration depth, and polarization properties in tissues. Their accuracy has been experimentally validated using tissue-mimicking phantoms, demonstrating strong agreement between simulated and measured results, reinforcing their reliability in capturing the complex light interactions in turbid media.

In this study, we apply a polarization-sensitive Monte Carlo simulation framework to model the propagation of polarized light through brain tissue with varying structural and optical characteristics. Specifically, we construct two representative models: one mimicking fiber bundles crossing in white matter, and another representing inclusion of optically isotropic tumor within optically anisotropic white matter of tumorless brain.

We analyze the trends of polarimetric observables, namely, linear retardance, depolarization, and optical axis orientation, across different depths and optical parameters, including birefringence strength and scattering coefficient. Through this investigation, our objective is to identify robust polarization signatures that can assist in distinguishing brain tumors from tumorless tissue under realistic intra-operative conditions.

## 2. Polarimetric set-up and brain tissue images

### 2.1. Wide-field imaging Mueller polarimeter

The imaging Mueller polarimeter (IMP) used in our experiments is a custom-designed, wide-field multi-spectral instrument, described in details in previous publications [28–31]. For the sake of completeness, we recall the main characteristics of the IMP. It makes use of electrically driven ferroelectric liquid crystals (FLCs) to achieve fast polarization modulation and filtering of probing and backscattered light beams, respectively, thus enabling the acquisition of 16 intensity images by the camera in just a few seconds. The IMP system operates in the visible wavelength range, using an incoherent white LED source. In order to avoid specular reflection, the illumination arm is mounted at $∼15^{\circ }$ to the axis of the camera, which is placed perpendicular to the sample surface.

The optimal configurations for polarization state generator (PSG) and polarization state analyzer (PSA) are chosen by maximizing the condition numbers of the corresponding modulation and analysis matrices as described in [32]. The calibration of the instrument was performed using the eigenvalue calibration method [33] by measuring an air and set of reference optical elements such as polarizers and a wave plate, in order to determine the real experimental configurations for PSG and PSA. The calibration enables pixel-wise reconstruction of the Mueller matrix of the sample from the 16 raw intensity measurements, corresponding to four input and four output polarization states, recorded at each wavelength of 500, 550, 600, and 650 nm using the corresponding bandpass filters.

### 2.2. Lu-Chipman decomposition of Mueller matrix

To extract physically interpretable polarimetric properties from a measured Mueller matrix **M** we used the polar decomposition suggested by Lu and Chipman [34]. Such decomposition of a physically realizable Mueller matrix represents a nonlinear data compression algorithm, which separates the complex effects of light interaction with matter described by the Mueller matrix into three basic optical properties: diattenuation, retardance, and depolarization. The Lu-Chipman decomposition of Mueller matrix **M** is expressed mathematically as:

(1) $$\text{M}={\text{M}}_{\Delta }{\text{M}}_{R}{\text{M}}_{D},$$

where ${\text{M}}_{D}$ , ${\text{M}}_{R}$ and ${\text{M}}_{\Delta }$ are the Mueller matrices of diattenuator, retarder, and depolarizer, respectively [35]. The list of polarimetric properties of bulk tissues measured in reflection at normal incidence (the most relevant configuration in clinical settings) includes i) strong depolarization due to light scattering, ii) moderate linear birefringence with almost non-existing circular birefringence and iii) very weak diattenuation (both linear and circular) [36]. Hence, ${\text{M}}_{R}$ represents the Mueller matrix of a linear retarder.

The total depolarization of a sample is calculated using

(2) $$\Delta =1-\frac{1}{3}\left|\mathrm{Tr}\left(M_{\Delta }-1\right)\right|,$$

where $M_{\Delta }$ represents the depolarization matrix obtained from Eq. (1). A value of Δ=1 corresponds to an ideal depolarizer, whereas Δ=0 holds for a non-depolarizing sample.

Linear retardance represents the phase shift between the eigen components of polarized light propagating through a uniaxial birefringent material. So called "form birefringence" of biological tissues is related to the presence of aligned collagen fibers, nerve bundles, etc. The scalar linear retardance *R* is computed as

(3) $$R=\arccos\left(\sqrt{r}-1\right)\;\text{and}\;r={\left(m_{R}^{\left(2,2\right)}+m_{R}^{\left(3,3\right)}\right)}^{2}+{\left(m_{R}^{\left(3,2\right)}-m_{R}^{\left(2,3\right)}\right)}^{2}$$

The azimuth of the optical axis θ of a linear birefringent medium contains the directional information on the optical anisotropy of a medium and is calculated as

(4) $$\theta =\frac{1}{2}\tan^{-1}\left(\frac{m_{R}^{\left(2,4\right)}}{m_{R}^{\left(4,3\right)}}\right),$$

where $m_{R}^{\left(i,j\right)}$ denotes the element of $M_{R}$ at the *i*-th row and *j*-th column. It was shown that pathological zones of tissues often demonstrate changes in both depolarization and linear birefringence, thus, suggesting the use of the polarimetric parameters for tissue differentiation [30,37–40].

### 2.3. Polarimetric images of tumorless brain tissue

The wide-field Mueller matrix images of a thick section (∼1 cm) of formalin-fixed human brain specimen from the autopsy of an anonymous donor were obtained with the IMP described in Sec. 2.1. The specimen was formalin-fixed but not embedded in paraffin. The fixation was performed in 4% formalin prepared in phosphate-buffered saline (PBS), with a pH of 6.9. The specimen was rinsed with distilled water prior to imaging. A waiver for ethical approval was obtained from the Ethics Committee of the Canton of Bern (BASEC-Nr: Req-2021-01173).

**Fig. 1.**
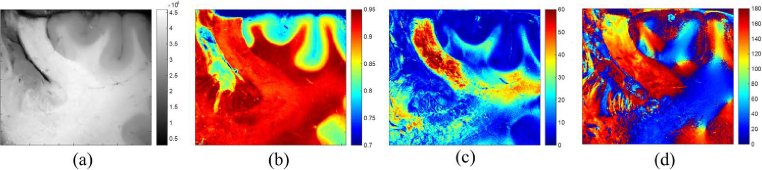
Wide-fied images of a thick section of formalin-fixed tumorless human brain: a) total reflected intensity (grey scale, a.u.); b) total depolarization (dimensionless); c) scalar linear retardance (degrees); d) azimuth of the optical axis (degrees).The size of the images is 3 cm × 2.5 cm, the measurement wavelength is 600 nm.

The corresponding maps of depolarization, scalar linear retardance and azimuth of optical axis (see Fig. 1) were obtained by applying pixel-wise polar Lu-Chipman decomposition described in Sec. 2.2. The specimen shown in Fig. 1 includes the gyrus cinguli and the corpus callosum, with adjacent white matter visible at the bottom of the images. The contrast between grey and white matter of brain is clearly seen in the images of both intensity and total depolarization. The values of depolarization are quite high (∼0.9) and uniformly distributed in the central Y-shaped zone (Fig. 1(b)), the values of linear retardance demonstrate significant drop in the bottom part of the image (Fig. 1(c)), while the orientation of the optical axis is not completely randomized there (Fig. 1(d)). Brain fiber bundles possess complex 3D architecture. When imaging a flat surface of tumorless brain cut shown in Fig. 1, we project this complex structure on a 2D imaging plane. The crossing and inclination of brain fiber bundles with respect to the imaging plane may affect locally the images of scalar retardance and azimuth of the optical axis potentially mimicking the expected optical isotropy of brain tumor zone, thus hindering the delineation of brain tumor borders.

## 3. Photon time of flight (PTOF) measurements of brain tissue

### 3.1. Instrumental set-up

White matter of brain tissue is known to be a negative linear birefringent medium [41] with the optical axis aligned with the direction of myelinated nerve bundles. Thus, one may expect that the speed of extraordinary ray propagating along the brain fiber bundles will be faster compared to the speed of ordinary ray propagating across the fiber bundles.

**Fig. 2.**
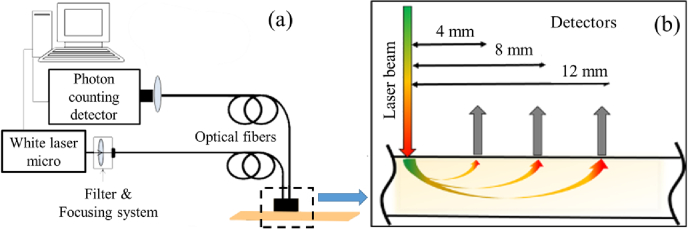
PTOF measurement setup: a) optical layout; b) schematics of laser beam injection point and different lateral positions of a detector at varying separation distance.

We implemented the photon time-of-flight (PTOF) optical modality, which represents a time-resolved measurement method, to estimate these two speeds in a white matter fiber bundles of fresh cadaveric calf brain. The main principle of PTOF is to deliver a very short laser pulse into the sample, and to detect and analyze the resulting pulse at some distance from the light injection point with the time resolution of picoseconds. To conduct such experiment the time-correlated single photon counting method [42] was used for optical pulse shape measurements (see Fig. 2).

A broadband picosecond laser (SuperK EVO EUL-10, 0.3W power, 60mW in VIS,425-2400 nm, 30MHz repetition rate, collimated) was used as the light source. Specific narrow spectral band was selected by the pair of identical interference filters : the first one was filtering the input light, while another was placed in front of the photo-detector (photomultiplier HPM-100-07 combined with the detector controller DCC-100 and data processing card SPC-150, Becker&Hickl GmbH, Germany). The examined spectral range was 600 — 800 nm; the spectral bands were selected with a 40 nm step using 10 nm half-bandwidth interference filters (Andover Corporation, USA - part numbers 600FS10-12,5; 640FS10-12,5; 680FS10-12,5; 720FS10-12,5; 760FS10-12,5; 800FS10-12,5).

Stable recording of optical signals via the input and output fibers (WF-400, Light Guide Optics International, LV, silica core diameter 400 microns, length 1.05 m) was ensured by means of a custom-made fiber holding probe with inter-fiber distance of 4 mm, 8 mm and 12 mm (see Fig. 2(b)). Before measurements, the input pulse shape (or instrumental response function IRF) was recorded by positioning the emitting and receiving fibers face-to-face utilizing a neutral density (ND) filter to avoid direct intense laser pulse impact on the detector. It determined the time scale for further measurements.

### 3.2. Measurements of corpus callosum of fresh calf brain

**Fig. 3.**
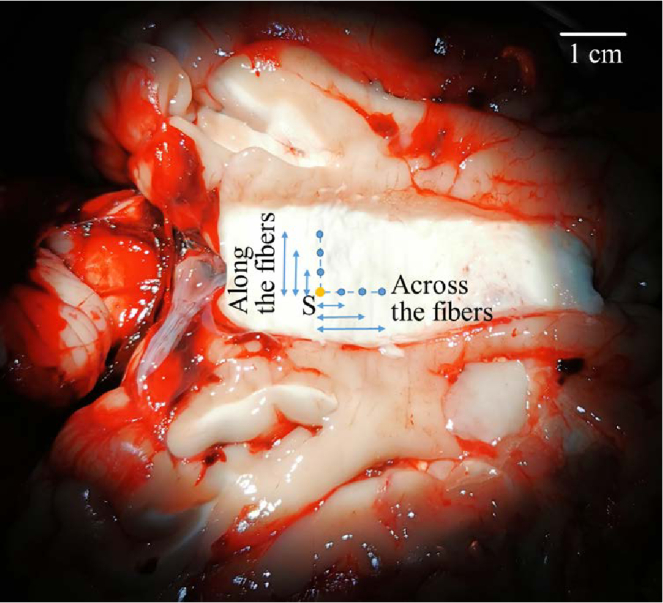
Top view of the open corpus callosum of a fresh calf brain (white zone in the image). S - point of laser beam injection. Blue points illustrate the detector location during the measurements along / across fiber bundles of excised corpus callosum.

We opened a corpus callosum of fresh calf brain bought from the local butchery, by moving apart two brain hemispheres (see Fig. 3). Corpus callosum represents a large tract of myelinated nerve fibers that enables communication between two hemispheres of a brain [43]. We excised the corpus callosum and performed two sets of PTOF measurements - along and across the fiber bundles - at different separation distances from the source to the detector.

**Fig. 4.**
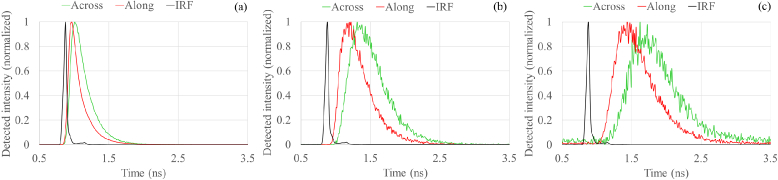
PTOF measurements of laser pulse propagation through corpus callosum tissue along / across nerve fiber bundles for the inter-fiber distance of a) 4 mm; b) 8 mm; c) 12 mm. The measured time difference between the mean amplitude of the main peak varies from 0.04 ns for 4 mm to 0.3 ns for 12 mm.

The results of PTOF measurements at 640 nm are shown in Fig. 4. The pulse travels faster along the fiber bundles, as expected for the extraordinary ray in a negative linear birefringent medium.

This phenomenon was observed at all measurement distances and all explored wavelengths. White matter of brain is not only birefringent, but is also multiple scattering medium. The widening of the detected pulse with increasing detector distance is due to a higher contribution from multiple scattering, which increases the path length of the detected photons.

To disentangle the impact of scattering and birefringence, we simulated the wide-field backscattered Mueller matrix images of brain-mimicking scattering and linear birefringent optical phantoms using polarized Monte Carlo algorithm. Details on the model derivation and the solution of the vector radiative transfer equation in scattering and optically anisotropic media are provided in the following section.

## 4. Model derivation and Monte Carlo solution algorithm

### 4.1. Vector radiative transfer equation

According to energy conservation law, the interaction of a polarized light beam with a disperse medium can be described by the solutions of the linear first order partial integro-differential vector radiative transfer equation(VRTE):

(5) $$\frac{1}{v}\frac{\partial L(r,\hat{\Omega },t)}{\partial t}+\hat{\Omega }\cdot \nabla L(r,\hat{\Omega },t)=-\mu_{e}L(r,\hat{\Omega },t)+\int_{\Omega \prime }\mu_{s}P({\hat{\Omega }}^{\prime }\to \hat{\Omega })L(r,{\hat{\Omega }}^{\prime },t)\;d\Omega \prime +\Sigma (r,\hat{\Omega },t)$$

where r describes the position within the scattering medium, *t*-time, L -vectorial radiance that is composed of the components of Stokes vector, *c* - speed of light in the vacuum, $v=c/n_{m}$ is the speed of light within the medium with the refractive index $n_{m}$ . The light beams can be deflected from their initial directions $\hat{\Omega }\prime$ and scattered into solid angle dΩ around the direction defined by the unit vector $\hat{\Omega }$ . The vector phase function $P(\hat{\Omega }\prime \to \hat{\Omega })$ is an angular-dependent 4×4 real-valued matrix that also depends on input vectorial radiance L because of normalization conditions, $\mu_{e}$ and $\mu_{s}$ are extinction and scattering coefficients, both of which are considered as constants Eq. (5). can be written as

(6) $$\frac{1}{v}\frac{\partial L(r,\hat{\Omega },t)}{\partial t}+\hat{\Omega }\cdot \nabla L(r,\hat{\Omega },t)+\mu_{e}L(r,\hat{\Omega },t)=q(r,\hat{\Omega },t)$$

where $q(r,\hat{\Omega },t)=\int_{\Omega \prime }\mu_{s}P(\hat{\Omega }\prime \to \hat{\Omega })L(r,\hat{\Omega }\prime ,t)\;d\Omega \prime +\Sigma (r,\hat{\Omega },t)$ - total rate at which photons appear at point $\left(r,\hat{\Omega },\text{t}\right)$ as the result of collisions and internal and external sources.

Equation (6) can be converted into an integral equation by the method of characteristics [44]. Assuming that there is only external source of photons, the VRTE in integral form can be written as

(7) L=KL+Q′

where K is the integral operator. We will consider the solution of Eq. (7) obtained by iterations

(8) $$L_{0}=Q\prime$$

(9) $$L_{1}=K\cdot L_{0}$$

⋮

(10) $$L_{n}=K\cdot L_{n-1}$$

It can be shown that at certain conditions on K and Q′ this iterative process converges to the solution of Eq. (7). Estimation of the integral of collision K is performed by the Monte Carlo technique, presented in the next subsection.

### 4.2. Monte Carlo solution of the VRTE

#### 4.2.1. Multiple scattering

It was demonstrated that analytical solution to the problem of scattering of plane electromagnetic (EM) wave on a single dielectric spherical scatterer exists and many asymptotic approximations of this solution are well studied. Contrary to that EM scattering by very large random group of particles occupying a volume in space is a problem of enormous complexity. The individual EM scattering field created by a particle in response to the EM scattering field of other particles can be comparable with the field created in response to the incident EM field. This means that single scattering approach is no longer valid. Therefore, we make several important physical assumptions [45]:

1. Each particle is located in the far field zones of all other particles; the detector is also located in the far-field zones of all scattering particles. Then, spherical wave created by a single scatterer in response to the incident plane wave can also be considered as a plane wave when it reaches the next particle. At least the mean distance between the scatterers has to be much larger than their radius and wavelength of incident light.
2. At any space point the total electric field can be represented as sum of contributions from light-scattering paths going through all possible particles sequences. All paths going through the same particle more than once can be neglected (Twersky approximation [46]). This assumption is justified when the total number of scattering particles in a volume is very large.
3. Full ergodicity of the system, i.e. the time-averaging of random object can be replaced by ensemble averaging (over position and state of the object).
4. The position and state of each particle are independent of the positions and states of all other particles. The spatial distribution of particles in the host medium is random.

To solve the VRTE in multiple scattering media (like biological tissue) using the Monte Carlo technique requires a stochastic model, where the mean values of certain random variables are the values of the physical properties (in our case the Mueller matrix coefficients of the scattering medium) to be determined by means of the numerical simulations. These mean values are calculated by averaging the multiple series of the independent samples.

The complete description of the Monte Carlo algorithm used in our studies can be found in [47,48]. For the sake of completeness the main steps of the algorithm are listed below.

• **Photon Initialization**: Generate a source photon with a defined position, propagation direction, and initial polarization state described by the Stokes vector $S^{in}$ .
• **Event Determination**: Identify the location and nature of the next interaction—such as scattering by a particle or reflection/transmission at an interface. If the photon reaches the simulation domain boundary, proceed to the step **Photon Termination**.
• **Photon Update**: Adjust the photon’s propagation direction and polarization based on the interaction encountered by multiplying Stokes vector associated with the photon by Mueller matrix of interface or Mueller matrix of scattering on spherical particle, respectively. The latter matrix is calculated using the exact Mie solution for spherical scatterer [49]. To speed up the calculation the coefficients of scattering matrix were pre-calculated and tabulated for the scattering angles varying between $0^{\circ }$ and $180^{\circ }$ with a step of $0.02^{\circ }$ .
• **Scoring and Continuation**: Record relevant data on the detector and verify whether the photon remains within the simulation domain. If it does, go to the step **Event Determination**.
• **Photon Termination**: The photon’s path ends once it exits the simulation domain, indicating no further interactions are possible.

The photon displacement at each step is calculated as $\Delta d=\min(d_{i},d_{s})$ , where $d_{i}$ is the distance till the closest interface along the direction of photon propagation, $d_{s}$ is the distance till the next scattering event defined as

(11) $$d_{s}=\frac{-\ln\zeta }{\mu_{e}},$$

where ζ is a pseudo-random number uniformly distributed over (0,1] and the extinction coefficient $\mu_{e}$ is the sum of the scattering coefficient $\mu_{s}$ and the absorption coefficient $\mu_{a}$ . If $d_{i}<d_{s}$ the Fresnel laws are applied at the interface, otherwise the photon undergoes scattering event. The statistical sampling of input Stokes vectors at the surface of Poincaré sphere is described by

(12) $$S^{in}(\theta ,\psi )={\left[1,\cos\;\theta ,\sin\;\theta \;\cos\;\psi ,\sin\;\theta \;\sin\;\psi \right]}^{T}$$

where θ and ψ are the polar and azimuthal angles on Poincaré sphere that vary over the intervals [0,π] and [0,2π] , respectively, according to a joint probability density function P(θ,ψ) . If we define correlation matrix D of the input components of Stokes vector as

(13) $$\text{D}=\int_{\theta =0}^{\pi }\int_{\psi =0}^{2\pi }S^{in}(\theta ,\psi )\cdot {\left[S^{in}(\theta ,\psi )\right]}^{T}P(\theta ,\psi )\;d\theta \;d\psi$$

and, similarly, correlation matrix G of the input and output components of Stokes vector as

(14) $$\begin{array}{ll} \text{G} & =\int_{\theta =0}^{\pi }\int_{\psi =0}^{2\pi }S^{out}(\theta ,\psi )\cdot {\left[S^{in}(\theta ,\psi )\right]}^{T}P(\theta ,\psi )\;d\theta d\psi \\ & =M\int_{\theta =0}^{\pi }\int_{\psi =0}^{2\pi }S^{in}(\theta ,\psi )\cdot {\left[S^{in}(\theta ,\psi )\right]}^{T}P(\theta ,\psi )\;d\theta d\psi =M\cdot D \end{array}$$

Mueller matrix M can be obtained from the correlation matrix G by the inversion of matrix D :

(15) $$\text{M}=\text{G}\cdot {\text{D}}^{-1}$$

It is obvious that existence of ${\text{D}}^{-1}$ depends on the choice of P(θ,ψ) . As we chose P(θ,ψ) to be a uniform distribution over the intervals [0, *π*] and [0, 2*π*], it gives a simple diagonal matrix D

(16) $$\text{D}=\pi^{2}\left[\begin{array}{cccc} 2 & 0 & 0 & 0\\ 0 & 1 & 0 & 0\\ 0 & 0 & 1/2\\ 0 & 0 & 0 & 1/2 \end{array}\right]$$

#### 4.2.2. Optical anisotropy

White matter of brain is not only scattering, but is also linear birefringent medium, largely due to the presence of densely packed nerve fiber bundles that induces so-called form birefringence. The spatial organization of brain fiber bundles determines the optical axis orientation, and their density influences the magnitude of polarimetric properties such as scalar linear birefringence. The phase velocity of light along the aligned fiber bundles differs from that perpendicular to the fiber bundles This difference produces a non-zero relative optical phase between the two orthogonal polarization components of the light. Phase retardation δ is defined as

**Fig. 5.**
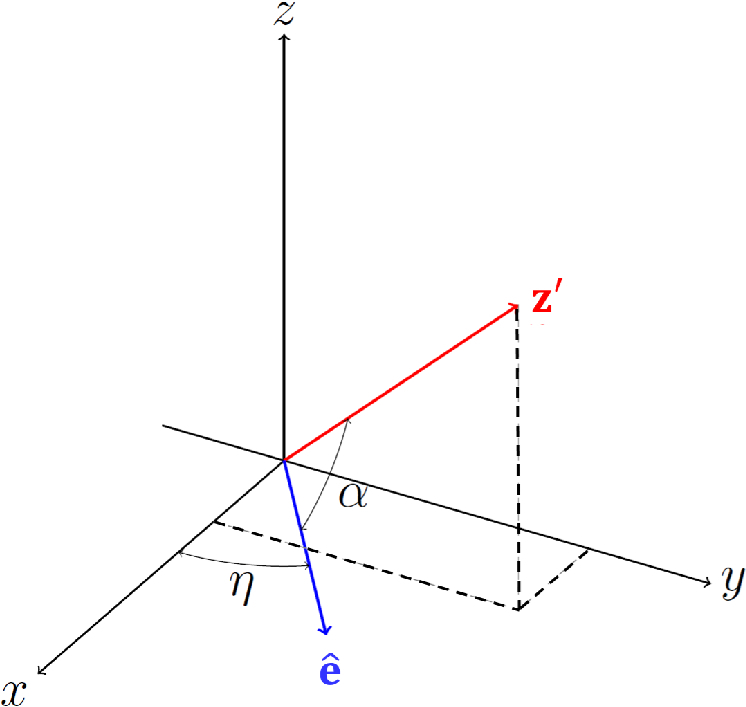
Vector of optical axis $\hat{\text{e}}(\cos\eta ,\sin\eta ,0)$ and the direction of light propagation ${\text{z}}^{\prime }(u_{x},u_{y},u_{z})$ in the laboratory coordinate system (x,y,z)

(17) δ=(2πdΔn)/λ

where d is the path length of light propagation through the uniaxial linear birefringent medium, and λ is the wavelength of probing light beam, Δn is defined as

(18) $$\Delta n=n(\alpha )-n_{o}$$

n(α) is the effective refractive index seen by light propagating at angle α with respect to the extraordinary axis of the uniaxial linear birefringent medium (see Fig. 5), and it is given by [50]

(19) $$n(\alpha )=\frac{n_{o}n_{e}}{\sqrt{n_{e}^{2}\cos^{2}\alpha +n_{o}^{2}\sin^{2}\alpha }},$$

where $n_{o}$ and $n_{e}$ are the ordinary and extraordinary refractive indices of the uniaxial linear birefringent medium, respectively.

The angle α between the vectors ${\text{z}}^{\prime }$ and $\hat{\text{e}}$ is defined from the scalar product:

(20) $$\alpha =\cos^{-1}\left(\frac{u_{x}\cos\eta +u_{y}\sin\eta }{\sqrt{u_{x}^{2}+u_{y}^{2}+u_{z}^{2}}}\right)$$

When the optical axis of the uniaxial linear birefringent medium is parallel to the x -axis, the Mueller matrix of linear retarder is given by

(21) $${\text{M}}_{R}=\left[\begin{array}{cccc} 1 & 0 & 0 & 0\\ 0 & 1 & 0 & 0\\ 0 & 0 & \cos\;\delta & \sin\;\delta \\ 0 & 0 & -\sin\;\delta & \cos\;\delta \end{array}\right]$$

To account for the phase shift of light propagating at the angle α with respect to the direction of the optical axis, we need to calculate the matrix product ${\text{M}}_{R}(\beta )=\text{R}(\beta ){\text{M}}_{R}\text{R}(-\beta )$ , where R(β) is the matrix of rotation in Stokes space:

(22) $$\text{R}(\beta )=\left[\begin{array}{cccc} 1 & 0 & 0 & 0\\ 0 & \cos2\beta & \sin2\beta & 0\\ 0 & -\sin2\beta & \cos2\beta & 0\\ 0 & 0 & 0 & 1 \end{array}\right]$$

**Fig. 6.**
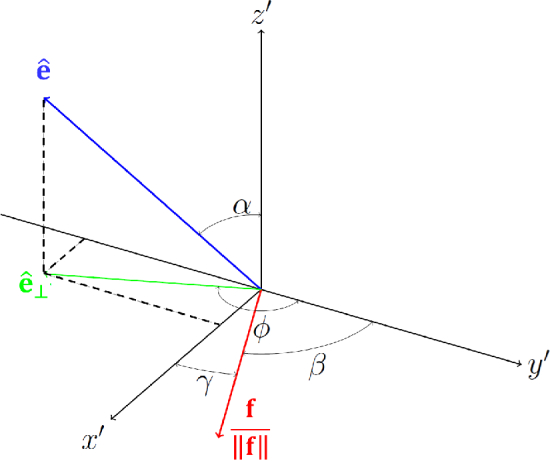
Vector of optical axis $\hat{\text{e}}$ in the local coordinate system: γ - the angle between the local optical axis and x′ -axis, ϕ - the angle between the orthogonal projection of the optical axis on x′−y′ plane and y′ -axis.

The rotation angle β is defined as the angle between the local optical axis $\text{f}=\text{k}\times \hat{\text{e}}$ (cross product of the vector of the direction of photon propagation z′=k and the vector of the optical axis $\hat{\text{e}}$ ) and unit vector of the y′ -axis of the local coordinate system (x′,y′,z′) (see Fig. 6). The rotation should always be performed counter-clockwise.

As photon propagates through a scattering linear birefringent medium, the angle α between the direction of optical axis and the direction of photon propagation z′ in the local coordinate system changes after each scattering event. Consequently, accurately determining both the local coordinate system and the local optical axis orientation is essential for reliable birefringence calculations. Thus, the updated polarized Monte Carlo algorithm for the solution of VRTE in scattering and optically anisotropic medium includes one extra step during the calculations of the trajectory of photon random walk, namely, the Stokes vector of a scattered photon is multiplied by the retarder matrix ${\text{M}}_{R}(\beta )$ , where parameter δ is calculated from Eq. (17) for the distance Δd traveled by a photon to the next scattering event site or to the boundary of birefringent medium.

## 5. Monte Carlo simulation results and discussion

### 5.1. Geometry and optical models of brain fiber bundles crossing and subsurface brain tumor inclusions

Our previous experiments revealed that the complex distribution of brain white matter fiber bundles causes a noticeable decrease in linear retardance [15] similar to that observed in tumors [40]. Therefore, we investigate whether it is possible to distinguish between these two different conditions based on maps of depolarization, linear retardance and azimuth of the optical axis, which is of great significance for the identification of tumor zones.

**Fig. 7.**
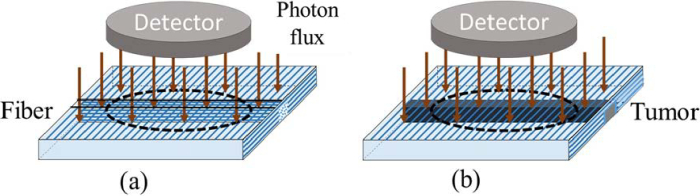
Optical phantom models used in Monte Carlo simulation of backscattered Mueller matrix images of brain tissue that include the subsurface volume to mimic: (a) fiber bundles crossing; (b) optically isotropic tumor.

We used Monte Carlo simulations to obtain the Mueller matrix images of scattering optically anisotropic phantoms shown in Fig. 7.

The scattering linear birefringent host medium (1 cm thick) contains an embedded subsurface parallelepiped made of the same scattering linear birefringent medium, but with its optical axis orientated orthogonally to that of the host medium to mimic the hidden crossing brain fiber bundles in the white matter of brain (Fig. 7(a)). We model a subsurface tumor inclusion by keeping the scattering properties of a medium within the parallelepiped, but just removing the linear birefringence within the tumor volume (see Fig. 7(b)).

By varying the distance from phantom surface to the top of crossing fiber bundle and tumor inclusion, we aim to explore the depth dependence of polarimetric parameters in the Mueller matrix images and corresponding polarimetric maps for both optical phantoms. By comparing the trends predicted by these two models, we can determine the polarization properties within the inclusion zones, and that will help us to differentiate them.

### 5.2. Parameters of model and data post-processing algorithm

A diffuse light source emitted the paquets of $10^{7}-10^{8}$ mono-energetic polarized photons (wavelength 633 nm) that impinged uniformly on the flat top surface of each phantom at normal incidence. The modulation of the photon polarization is described by Eq. (12). A two-dimensional detector grid consisting of 100×100 square pixels of 0.1 mm × 0.1 mm size was defined first. Then a circular detection region was defined by inscribing a 1 cm diameter circle within 1 cm × 1 cm array of pixels. Only the pixels entirely located within this circular area were included in the analysis, resulting in a circular-shaped active detection zone composed of a subset of the full grid. The circular detector was placed at the distance of 5 cm from the phantom surface to directly register backscattered photons (see Fig. 7).

Our prior studies of various tissue types with the wide-field imaging Mueller polarimetry demonstrated that in backscattering configuration the detected signal is dominated by scattering on particles that are much smaller than the wavelength of probing light (so-called Rayleigh regime) [30,39]. Hence, the impact of the exact shape of the particle on scattered electromagnetic field is negligible. In our optical phantoms we defined five group of spherical scatterers with the size defined by the Gaussian distribution centered at 50 nm to account for the natural variability of scattering properties of biological tissues.

The particle number density for each group was selected to ensure the average scattering coefficient for the white matter of healthy brain $\mu_{s}=400{\text{ cm}}^{-1}$ [51], resulting in a mean free path $l_{s}={\mu_{s}}^{-1}=25\;\mu \text{m}$ . At this stage there was no absorption taken into account $\left(\mu_{a}=0\right)$ . The birefringence of a host medium was characterized by the refractive index difference of $\Delta n=n_{e}-n_{o}=-10^{-4}$ , with $n_{e}=1.33$ and the refractive index of scattering particles $n_{p}=1.59$ . The azimuth of the optical axis of a host medium was set at $90^{\circ }$ , the azimuth of the optical axis of crossing fiber bundle was set at $0^{\circ }$ in the laboratory coordinate system.

Using Eqs (12–16) the backscattered complete Mueller matrix images of the optical phantoms were obtained, ranging from $m_{11}$ to $m_{44}$ . All elements of the Muller matrix images $m_{ij}^{*}(k,m)$ were normalized by $m_{00}(k,m)$ pixel-wise: $m_{ij}^{*}(k,m)=m_{ij}(k,m)/m_{11}(k,m)(k,m=1,\ldots ,100;\;i,j=1,\ldots ,4)$ . Because of the statistical nature of Monte Carlo algorithm the convergence criterion was set: the maximum of standard deviation of the normalized coefficients $m_{ij}^{*}(k,m)$ should be less than 1.5% .

To ensure the physical realizability of the simulated Mueller matrices, the Cloude filtering [52] was applied to exclude the pixels with non-physical Mueller matrices. The maps of depolarization, scalar linear retardance and azimuth of the optical axis were generated by applying pixel-wise Lu–Chipman decomposition of the simulated Mueller matrix images.

### 5.3. Brain tissue phantoms: trends in polarimetric parameter values with depth

**Fig. 8.**
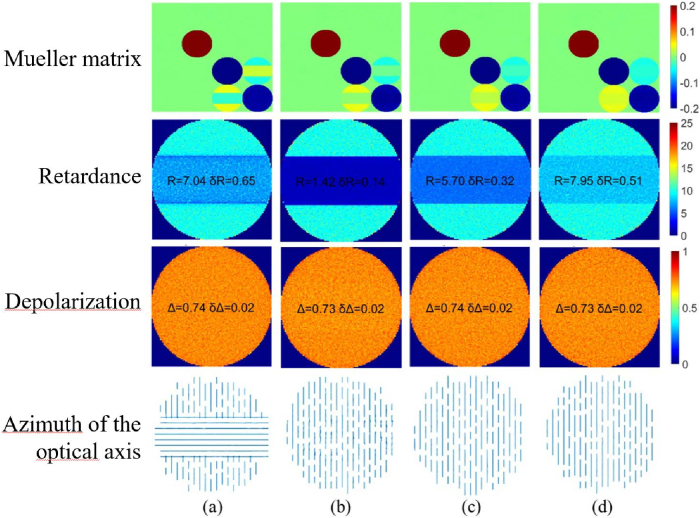
Simulated Mueller matrix images and corresponding maps of linear retardance (degrees), depolarization (dimensionless) and quiver plot of the azimuth of the optical axis for (a) crossing fiber bundles and (b) tumor inclusion placed at the depth of $l_{s}$ ; (c) and (d) - the analogous images at the depth of $15l_{s}$ . Mean value and standard deviation for the inclusion zone are shown in the images of linear retardance and depolarization.

We simulated the Mueller matrix images of both optical phantoms at different depths of the fiber bundle and tumor inclusion. Figure 8 shows the example of simulated Mueller matrix images, the corresponding maps of linear retardance, depolarization, and azimuth of the optical axis for two optical phantoms (see Fig. 7) at different inclusion depths $l_{s}$ and $15l_{s}$ .

The retardance image contrast between the host medium and the subsurface crossing fiber bundle or tumor inclusion zones is higher at both depths compared to the same contrast in the depolarization image. It is worth mentioning that the scattering coefficient $\mu_{s}$ was kept the same for both optical phantoms. Despite the detectable difference in the mean values of linear retardance for the crossing fiber bundle inclusion zone (Figs 8(a),c), the azimuth of the optical axis of crossing fiber bundle is not detectable at the immersion depth of $15l_{s}$ . The same trends for the scalar linear retardance and azimuth of the optical axis were observed experimentally, when measuring superimposed thin sections of formalin-fixed human brain corpus callosum tissue of varying thicknesses [53].

**Fig. 9.**
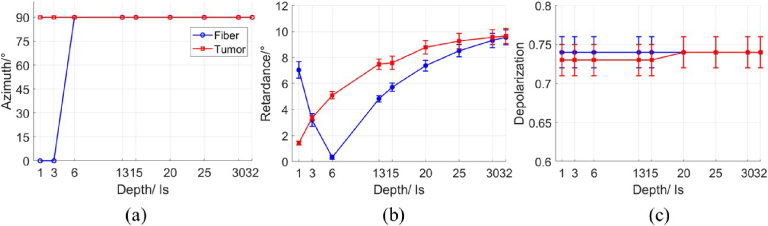
Depth dependence of (a) azimuth of the optical axis (degrees); (b) linear retardance (degrees); (c) depolarization (dimensionless). Depth is expressed in $l_{s}$ units.

We also simulated the Mueller matrix images and corresponding polarimetric maps of the linear retardance, depolarization and azimuth of the optical axis for the optical phantoms with crossing fiber bundle and tumor inclusion placed at different depths. Figure 9 illustrates the depth dependence of the polarimetric parameters averaged over the inclusion zones for both phantom models. A transition occurs at depth $∼6l_{s}$ in the graphs of the orientation of the optical axis and the linear retardance of the optical phantom with crossing fiber bundles. The zero-degree azimuth of the optical axis in the hidden fiber bundle zone is replaced by the 90-degree azimuth of the host medium. The azimuth of 90^∘^ remains constant with increasing depth of embedded fiber bundle. The value of scalar retardance drops to almost zero when the top surface of the crossing fiber bundle reaches a depth of $6l_{s}$ . This means that the retardance introduced by the top layer of the host medium is almost completely compensated by the retardance introduced by a hidden fiber bundle with the orthogonal orientation of the optical axis.

Linear retardance values increase monotonously with depth for the optical phantom with tumor inclusion because the thickness of the top linear birefringent layer increases. The linear retardance values became equal for both optical phantoms at depth $∼32l_{s}$ (i.e. ∼0.8 mm). Obviously, the azimuth of the optical axis for the optical phantom with tumor remains constant with depth and equal to that of the host medium. At depth $∼20l_{s}$ (i. e. 0.5 mm) the depolarization values for the phantom with tumor inclusion start to become smaller compared to the depolarization values for the phantom with crossing fiber bundles. The summary of the results is presented in Table. 1.

**Table 1.** Trends in polarimetric parameter values with the inclusion depth for two optical phantoms. T - top layer value, B - bottom layer value, ${\text{C}}_{1}$ and ${\text{C}}_{2}$ - constant values; ↓ - value decrease; ↑ - value increase

Crossing fiber bundles					Tumor inclusion			
Depth	$32l_{s}$	$20l_{s}$	$6l_{s}$	$l_{s}$	$32l_{s}$	$20l_{s}$	$6l_{s}$	$l_{s}$
Depolarization	${\text{C}}_{1}$	${\text{C}}_{1}$	${\text{C}}_{1}$	${\text{C}}_{1}$	${\text{C}}_{1}$	↓	${\text{C}}_{2}$	${\text{C}}_{2}$
Retardance	↓	↓	0	↑	↓	↓	↓	↓
Azimuth	B	B	B	T	B	B	B	B

The practical relevance of the simulation results can be interpreted in a surgical context. As a neurosurgeon removes progressively the superficial layers of brain tissue and approaches either tumor or fiber bundle crossing zone, the linear retardance starts to decrease at the distance of $∼32l_{s}$ (i. e. 0.8 mm in our model). If the detectable reduction in depolarization follows at $∼15l_{s}$ (i. e. ∼0.4 mm in our model), it suggests the presence of a tumor. In contrast, if a kink in linear retardance curve and a change in optical axis orientation are observed at $∼6l_{s}$ (i.e 0.15 mm in our model), the inclusion is likely to be a crossing fiber bundle. In the case of a crossing fiber bundle, the kink in the retardance curve appears at a shallower depth than the onset of the relative depolarization drop observed in a tumor zone. Therefore, the depolarization decrease may serve as the earliest optical indicator of an underlying tumor inclusion.

It is important to note that the optical properties of human brain tissue are inherently complex and vary across individuals and pathological conditions. Therefore, in the following section we will investigate the influence of important optical parameters, namely, phase retardation Δn and scattering coefficient $\mu_{s}$ derived from experimental data for human brain, on depolarization image contrast in tumor model.

### 5.4. Impact of optical anisotropy and scattering on depolarization contrast in optical model of brain tumor

We investigate how the variations in parameter Δn affect the depolarization contrast between tumor regions and surrounding anisotropic white matter of brain. Prior studies have shown that the maximum values of |Δn| for biological tissues can vary between $10^{-5}$ to $10^{-3}$ [54] under different physiological or pathological conditions.

We used the Monte Carlo algorithm described in Section 4.2 to simulate the Mueller matrix images and corresponding polarimetric maps for the optical model of tumor inclusion at depth $l_{s}$ . The value of the parameter Δn of the host medium was varied between $-10^{-4}$ and $-10^{-3}$ , while tumor inclusion was modeled as optically isotropic medium. The scattering coefficient $\mu_{s}$ was kept $400\;{\text{cm}}^{-1}$ for both host medium and tumor inclusion.

**Fig. 10.**
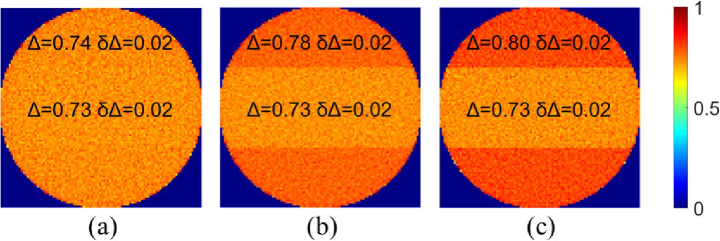
Maps of the depolarization for the optical model of tumor inclusion at depth $l_{s}$ for (a) $\Delta n=-10^{-4}$ ; (b) $\Delta n=-5\times 10^{-4}$ ; (c) $\Delta n=-10^{-3}$

The simulation results shown in Fig. 10, indicate that increasing |Δn| leads to a higher contrast between the tumor zone and surrounding tissue in the maps of depolarization.

It proves that linear birefringence contributes significantly to depolarization in optically anisotropic tissues, The difference in corresponding averaged values of depolarization varies from ∼1% for $\Delta n=-10^{-4}$ to ∼9% for $\Delta n=-10^{-3}$ . The variations in depolarization values above 1−2% can be accurately detected with a modern Mueller polarimetric systems [29]. These findings underscore the crucial role of linear birefringence in improving the contrast in depolarization image between optically isotropic tumor zone and strongly birefringent host tissue.

The scattering coefficient of tumor inclusion is another important parameter of the optical model. Prior studies have shown that different types and stages of brain tumors exhibit typically lower scattering coefficients compared to tumorless brain tissue [51,55,56]. We investigate the impact of varying tumor scattering coefficient $\mu_{s}^{t}$ on the contrast between tumor zone and host medium in the maps of depolarization.

**Fig. 11.**
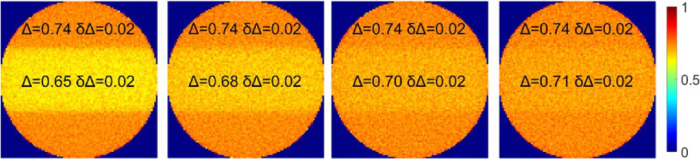
Maps of the depolarization for the optical model of tumor inclusion at depth $l_{s}$ for (a) $\mu_{s}^{t}=120{\text{ cm}}^{-1}$ ; (b) $\mu_{s}^{t}=171{\text{cm}}^{-1}$ ; (c) $\mu_{s}^{t}=240{\text{ cm}}^{-1}$ ; (d) $\mu_{s}^{t}=280{\text{ cm}}^{-1}$ . The scattering coefficient of the host medium was kept at $400{\text{ cm}}^{-1}$ .

We set the scattering coefficient of tumorless brain white matter to $\mu_{s}=400{\text{ cm}}^{-1}$ and parameter $\Delta n=-10^{-5}$ . Four representative values of tumor scattering coefficients were selected: $\mu_{s}^{t}=120{\text{ cm}}^{-1}$ , $171{\text{ cm}}^{-1}$ , $240{\text{ cm}}^{-1}$ and 280 cm^−1^. Tumor inclusion was placed at depth $l_{s}$ . The simulated maps of depolarization are presented in Fig. 11. A lower value of $\mu_{s}^{t}$ results in higher contrast between tumor zone and surrounding tissue in depolarization maps, thus improving the detectability of a tumor. A maximum contrast of ∼7% is observed for $\mu_{s}^{t}=120{\text{ cm}}^{-1}$ compared to the contrast of ∼4% , which is observed for $\mu_{s}^{t}=280{\text{ cm}}^{-1}$ . These results highlight the potential of using the combination of polarimetric maps of depolarization, scalar linear retardance and azimuth of the optical axis for the differentiation between the zones of tumor and crossing fiber bundles during brain tumor surgery.

## 6. Conclusion

In this study, we employed Monte Carlo algorithm of the solution of VRTE to investigate the polarization properties of optically anisotropic brain tissue and its alterations in the presence of complex brain fiber bundles distribution and optically isotropic tumor inclusions. By devising two optical models that mimic brain fiber bundles crossing and tumor inclusion, we analyzed the impact of each inclusion depth variation on the values of polarimetric parameters, namely, linear retardance, orientation f the optical axis and depolarization. The linear retardance values are sensitive to the presence of any type of inclusion at depth $32l_{s}$ (∼ 0.8 mm in our model). However, the trends for linear retardance value with depth decrease are similar for both crossing fiber bundles and tumor zones. On contrary, the trends in depolarization values with depth decrease are different starting from depth $15l_{s}$ (∼ 0.4 mm in our model). The change in the azimuth of the optical axis for the crossing fiber bundles model manifests itself at an even shallower depth of $6l_{s}$ (∼ 0.15 mm in our model).

Changes in values of parameter |Δn| of the host medium and scattering coefficient $\mu_{s}^{t}$ of a tumor demonstrate the increase of contrast between tumor zone and host medium in the images of depolarization with the increase of |Δn| and the decrease of tumor scattering coefficient $\mu_{s}^{t}$ .

Our results demonstrate that the combination of the maps of depolarization, linear retardance and azimuth of the optical axis may enable the reliable discrimination between tumorless brain white matter tissue and tumor zones. It is worth mentioning that the calculated maps of the azimuth in Fig. 8 show the orientation of the optical axis. This is consistent with the parameters of our optical model of negative linear birefringent white matter of brain, where the extraordinary refractive index was set to be smaller than the ordinary one. It is not possible to separate the impact of linear birefringence and scattering in PTOF experiments (see Sec. 3), however, we can disentangle these phenomena using both experimental and simulated Mueller matrix images and Lu-Chipman decomposition.

Neurodegenerative diseases, such as Alzheimer’s, may influence polarization-based imaging [57], and this should be taken into account when using wide-field IMP in patients with pre-existing neurocognitive conditions. However, in practical terms, tissue property variations due to neurodegeneration are unlikely to significantly impact IMP’s utility in glioma surgery, as low-grade gliomas predominantly occur in neurologically healthy young adults, while older patients with severe cognitive decline are often ineligible for surgery. Thus, although intra-operative IMP could potentially serve as a screening tool for local plaque formation or neurodegenerative changes - especially in older patients undergoing surgery for hydrocephalus or hemorrhages - these disturbances are unlikely to affect the overall efficacy of IMP during neurosurgery for brain tumors.

While our findings provide valuable optical insights, the complexity of real brain tissue necessitates further research to validate and expand upon these results. Incorporating a wider range of tissue types and optical parameters (e. g. $\mu_{a}\neq 0$ ) will help bridge the gap between simulations and clinical applications of wide-field IMP, ultimately contributing to the development of a new optical technique for more accurate tumor detection during neurosurgery. The main advantage of wide-field IMP lies in its potential for rapid intra-operative diagnostic use. Unlike conventional histological methods, which require extensive sample preparation and staining, Mueller polarimetry can provide structural information in real time, thereby, enabling quicker decision-making during surgical interventions.

## Data Availability

Data underlying the results presented in this paper are not publicly available at this time but may be obtained from the authors upon reasonable request.
